# Effects of early rehabilitation in sepsis patients by a specialized physical therapist in an emergency center on the return to activities of daily living independence: A retrospective cohort study

**DOI:** 10.1371/journal.pone.0266348

**Published:** 2022-03-31

**Authors:** Yasunari Sakai, Shuhei Yamamoto, Tatsunori Karasawa, Masaaki Sato, Kenichi Nitta, Mayumi Okada, Kanako Takeshige, Shota Ikegami, Hiroshi Imamura, Hiroshi Horiuchi

**Affiliations:** 1 Department of Rehabilitation, Shinshu University Hospital, Matsumoto, Japan; 2 Department of Occupational Therapy, School of Health Sciences, Shinshu University, Matsumoto, Japan; 3 Department of advanced emergency critical care center, Shinshu University Hospital, Matsumoto, Japan; Stanford University School of Medicine, UNITED STATES

## Abstract

**Background:**

Early rehabilitation allows patients to better perform the activities of daily living after hospital discharge. A specialized physical therapist has been assigned as part of the early rehabilitation, but the effectiveness of the program remains unclear. We investigated how early rehabilitation provided by a specialized physical therapist affects ADL in patients with sepsis.

**Methods:**

This was a retrospective cohort study. This study’s subjects were sepsis patients who entered the advanced emergency critical care center of Shinshu University Hospital between April 2014 and March 2020. Electronic medical records were reviewed to obtain information on demographic characteristics, severity score, primary source of infection, therapeutic medication, the number of days after hospital admittance until rehabilitation begins, length of hospital stay, discharge to home, and an assessment of daily living activities for each patient. The patients were divided into two groups based on whether they were treated before or after a specialized physical therapist had been hired by the advanced emergency critical care center.

**Results:**

Assigning a physical therapist to a patient significantly shortened the number of days until rehabilitation began. In a multivariable model, the strongest predictors of return to independent living after hospital discharge were (1) assigning a specialized physical therapist (odds ratio = 2.40; 95% confidence interval = 1.09–5.79; P = 0.050) and (2) the number of days until rehabilitation started (odds ratio = 0.24; 95% confidence interval = 0.08–0.76; P = 0.014).

**Conclusions:**

Assigning a specialized physical therapist to sepsis patients at an advanced emergency critical care center significantly shortened the number of days until a patient can begin rehabilitation after hospital admittance and improved activities of daily living after hospital discharge.

**Trial registration:**

Trial registration [University Hospital Medical Information Network Clinical Trials Registry, number UMIN000040570 (2020/5/28).]

## Background

Sepsis is a powerful systemic response to severe infection, and its incidence is estimated to be between 50 and 100 cases per 100,000 people in developed countries [1]. Sepsis patients have significantly worse functional outcomes in the physical and cognitive domains than age-matched healthy individuals [2] and other hospitalized patients [3]. A previous study showed that long-term cognitive and motor functions were significantly reduced in patients with severe sepsis compared to patients with mild or moderate sepsis [3].

Sepsis patients in the intensive care unit (ICU) can develop post-intensive care syndrome (PICS) [4], which is classified by a decline in physical, cognitive, or mental functions, and ICU-acquired weakness (ICU-AW) [5, 6]. ICU-AW occurs in 46% of critically ill patients with sepsis, multiple organ failure (MOF), or long-term ventilation [7]. These patients are often discharged to long-term physical rehabilitation facilities [8], which consume resources [9] and are costly [10]. It would be beneficial to develop strategies for sepsis patients to improve outcomes following hospitalization, and early rehabilitation interventions are preventive measures for PICS and ICU-AW.

In a previous study of 104 critically ill ICU patients, an early rehabilitation group (sedation interruption, extremity mobilization, and early activities of daily living training) was compared to a control group that had received standard care, using the Barthel Index (BI), which measures the capability to carry out the activities of daily living (ADL) (BI score: 75 points vs. 55 points, respectively); functional status was also significantly improved (59% vs. 35%) [11]. In addition, a large improvement in physical function was seen in a study of hospitalized patients who received early rehabilitation and active exercise at the bedside [12–14]. These previous studies, however, included patients with a wide range of conditions: postoperative, with acute lung injury, in the ICU, or on ventilators. Studying a wide variety of diseases diminishes specificity, and few studies have focused solely on the rehabilitation of sepsis patients.

In 2018, a randomized controlled trial studied long-term rehabilitation methods limited to sepsis patients [15], but it the only result was that a beneficial effect was obtained in severely ill patients. We have already treated patients based on the Awakening and Breathing Coordination, Delirium monitoring / management, and Early exercise / mobility bundle [16]. Following the example of other countries, early mobilization and rehabilitation in Japan has made considerable progress and is now being implemented in many facilities. Then More and more facilities are assigning full-time physical therapists for the purpose of increasing clinical effectiveness and clinical efficiency.

Our hospital is also a specialized physical therapist has been assigned since April 2017 at the advanced emergency critical care center (AECCC) as a part of the promotion early rehabilitation. However, there are no studies to date that have shown the effects of assigning a specialized physical therapist, and the dilution of the significance of assigning a specialized physical therapist has become a problem in society.

We hypothesized that having a specialized physical therapist would increase the facilitation of early rehabilitation and have a more beneficial effect on the ADLs of critically ill sepsis patients. Therefore, we investigated how early rehabilitation provided by a specialized physical therapist affects ADL in patients with sepsis.

## Methods

This was a retrospective cohort study. The outcome assessors and researchers were blinded to prevent bias. The information detailed below was collected from electronic medical records for each patient. In 2017, a specialized physical therapist was hired by the AECCC to promote early rehabilitation, and we compared outcomes from groups of patients treated before or after the specialist joined the staff. Patients in the treatment after assigning specialized physical therapist were admitted between April 2017 and March 2020, and patients in the before assigning specialized physical therapist were admitted from April 2014 to March 2017.

### Sample size

Sample size calculations used the G*power 3.1. A sample size of 64 per group (total 128) was calculated with an effect size of 0.5 and 80% power with a type 1 error rate of 0.05 with a Bonferroni adjustment.

### Patients

This study’s subjects were sepsis patients who entered the AECCC of Shinshu University Hospital between April 2014 and March 2020. Patients were included if they were 18 years or older, had received intensive treatment, and were diagnosed with sepsis (two or more criteria of a systemic inflammatory response plus proven or strongly suspected infection) [17], sepsis plus organ failure, or septic shock (sepsis with hypotension not responding to fluid management). To be enrolled, patients also needed to meet the criteria for baseline functional independence, defined a priori as a BI score ≥70, obtained from a proxy describing patient function before admission [18, 19]. Patients were excluded if they had head injuries, burns, spinal injuries, lower limbs with multiple fractures, septic shock unresponsive to treatment, or expected mortality within 48 hours. We recorded each patient’s age, gender, body mass index, sequential organ failure assessment score [20], systemic inflammatory response syndrome score [21], disseminated intravascular coagulation (DIC) score, laboratory data (procalcitonin), primary source of infection, use of therapeutic medication, use of mechanical ventilation, BI baseline and discharge, length of hospital stay, patients discharge outcome, and the number of days until rehabilitation after hospital admittance.

### Physical rehabilitation content of the AECCC

We recorded data from the physical therapy sessions of both groups. Participants in both groups received standard AECCC care, which included physical therapy rehabilitation. Participants in the intervention arm worked with the specialized physical therapist hired by the hospital in 2017 and each was given an individualized therapy plan for physical rehabilitation that started on average 2.7 days after admission to the hospital. The therapy ran once or twice daily, for 20–40 min per session, until discharge from the AECCC. The therapy included pulmonary rehabilitation (deep breathing, periodic noninvasive ventilation, and supported cough), active and passive ranges of motion in both upper and lower extremities, getting out of bed, transfer, electrical muscle stimulation (EMS) (General Therapeutic Electrical Stimulator; Homer Ion Co., Tokyo, Japan), ambulation, and other mobilization techniques. Therapeutic interventions were continued regularly throughout the patient’s hospital stay, until he or she returned to a previous function, and/or was discharged. A safety audit was completed on patients in the intervention group during treatment. Recently published literature recorded patients who suffered falls, endotracheal tube removal, systolic blood pressure over 200 mmHg, systolic blood pressure less than 90 mmHg, and oxygen desaturation less than 80% [22]. In this study, we focused on these adverse events and developed a rehabilitation program.

### Primary outcomes

Using the BI, participants were assessed objectively by the physical therapist for ADL recovery at discharge from the hospital [18, 19]. Using the parameters of previous studies, a BI of 70 points or more is defined as ADL independence [18].

### Secondary outcomes

Secondary measures included length of hospital stay and discharge outcome.

### Statistical analysis

Single variables were compared using a Student’s t test and a Mann-Whitney U test. Dichotomous outcomes were analyzed using a chi-square test. Moreover, we used logistic regression analysis to examine the relationship between therapy and return to independent ADL at hospital discharge. We performed multivariable analysis (logistic regression analysis) that included therapy by a specialized physical therapist and the number of days until rehabilitation began after hospital admittance. We used previous research as a reference to stratify the number of days until rehabilitation began (≤ five days vs. ≥ six days) [23, 24]. To avoid overfitting, we reduced all potential confounding factors, including treatment by the specialized physical therapist and the number of days until rehabilitation, to a single composite characteristic by applying a propensity score. The analyses were performed using the EZR statistical program (open-source software) [25]. Descriptive statistics (mean ± standard deviation or median [25%, 75%]) were calculated. All tests at P ≤ 0.05 were considered statistically significant.

### Ethics

This study met the requirements for appropriateness to achieve the intended aims and complied with all ethical principles pertinent to this type of study design. All data were fully anonymized before the analyses. No informed consent was required due to the characteristics of the design and we obtained an opt-out format was adopted for all participants. The study was approved by the Ethics Committee of Shinshu University (No, 4161) and registered with University Hospital Medical Information Network Clinical Trials Registry, number UMIN000040570 (2020/5/28). This study was conducted in accordance with the standards set forth by the latest revision of the Declaration of Helsinki.

## Results

**Fig 1:**
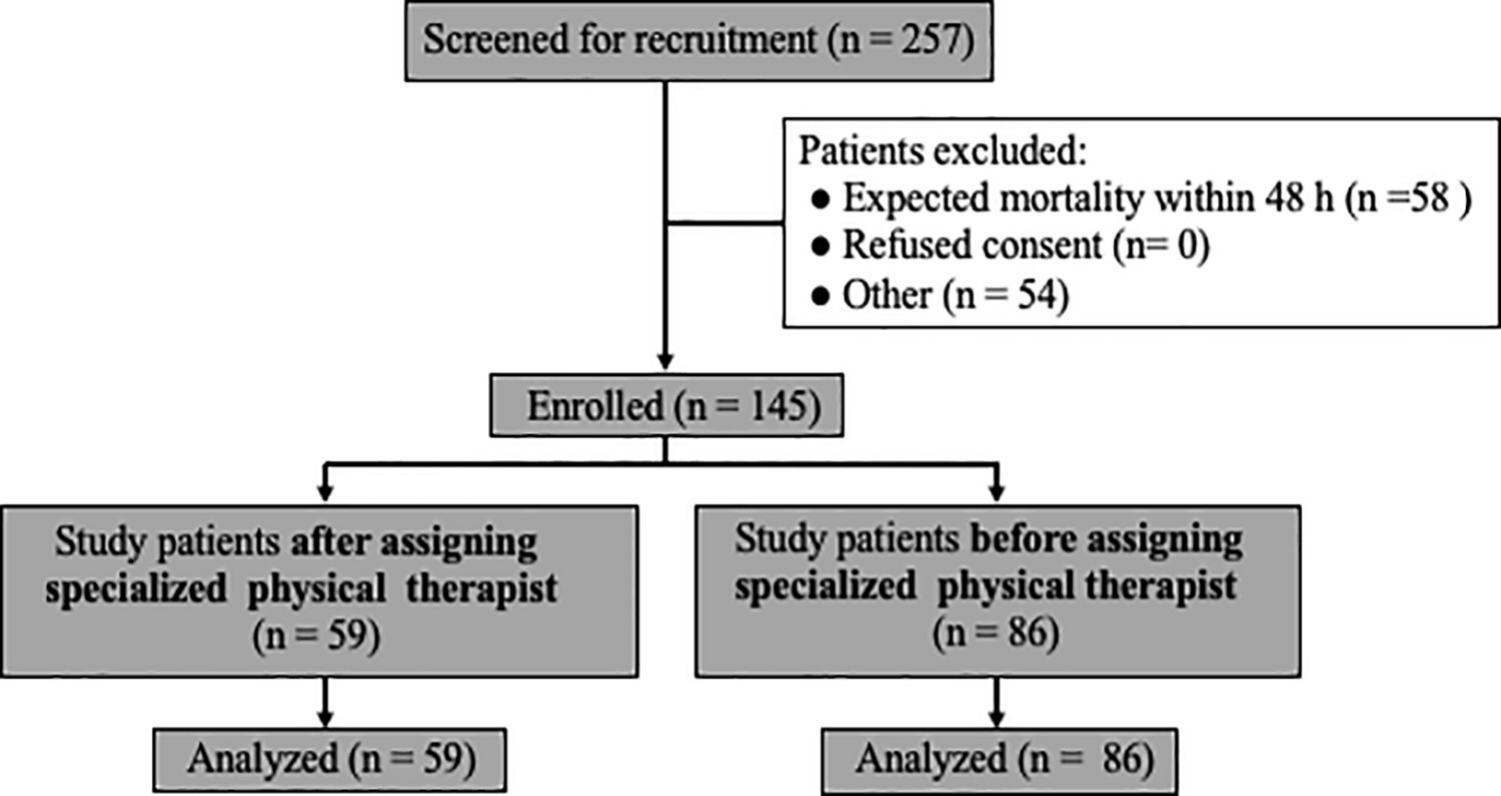
Flow chart of study participation. Two hundred fifty-seven subjects were in the initial patient pool. After exclusion criteria were applied, 145 subjects were included in the analysis.

In total, 257 patients were diagnosed with sepsis and were entered the AECCC of Shinshu University hospital between April 2014 and March 2020. Of 145 (56.4%) sepsis patients that met inclusion criteria during the study period. 86 patients between April 2014 and March 2017 and 59 patients were recruited between April 2017 and March 2020 (Fig 1). There were no adverse events related to rehabilitation observed in this study.

### Adherence

There were no withdrawals during the study. All participants adhered to the protocol and remained in the study for an average of 33 days.

### Demographics, AECCC, and hospital measures

**Fig 2:**
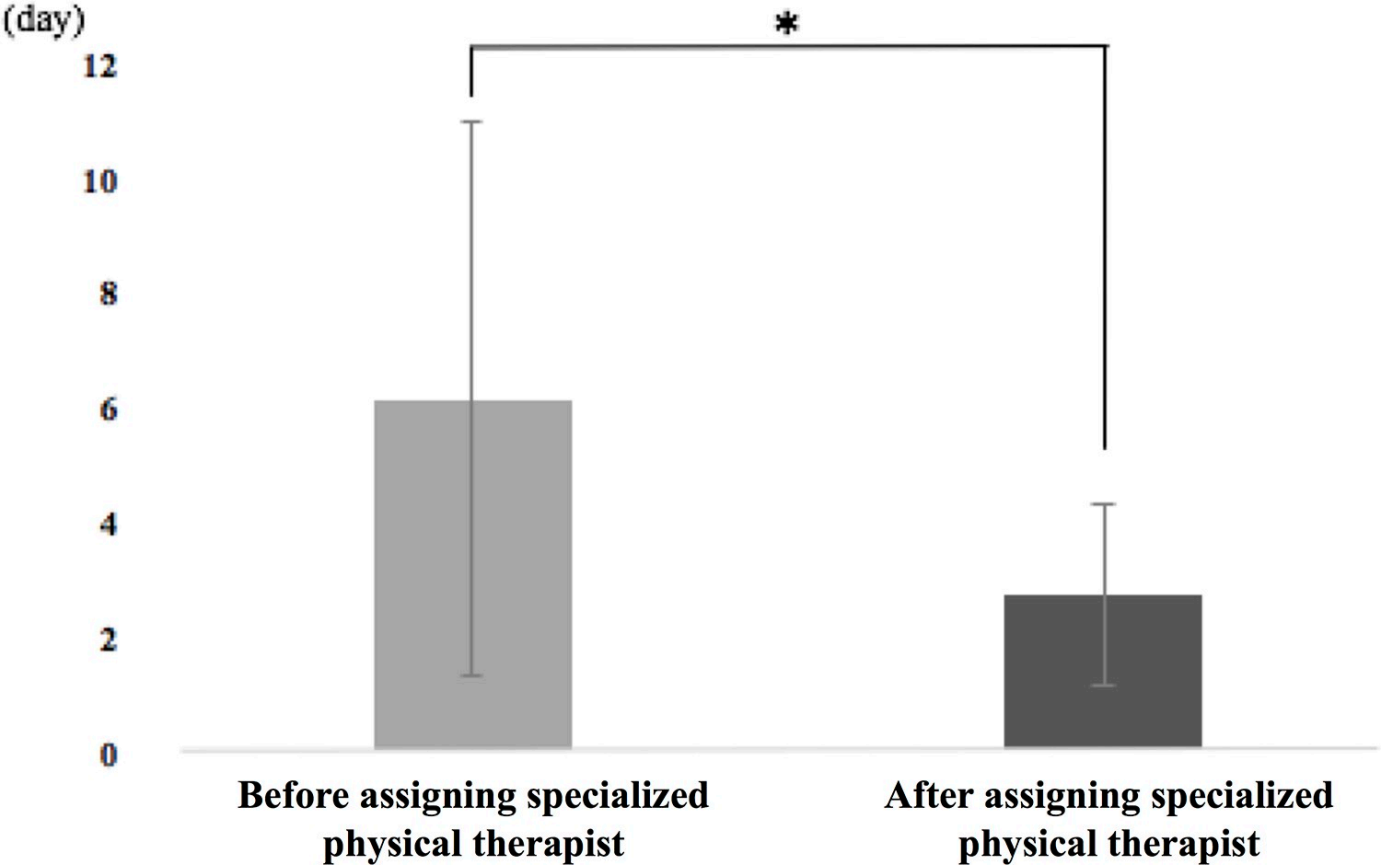
The number of days until rehabilitation in patients with sepsis.

**Fig 3:**
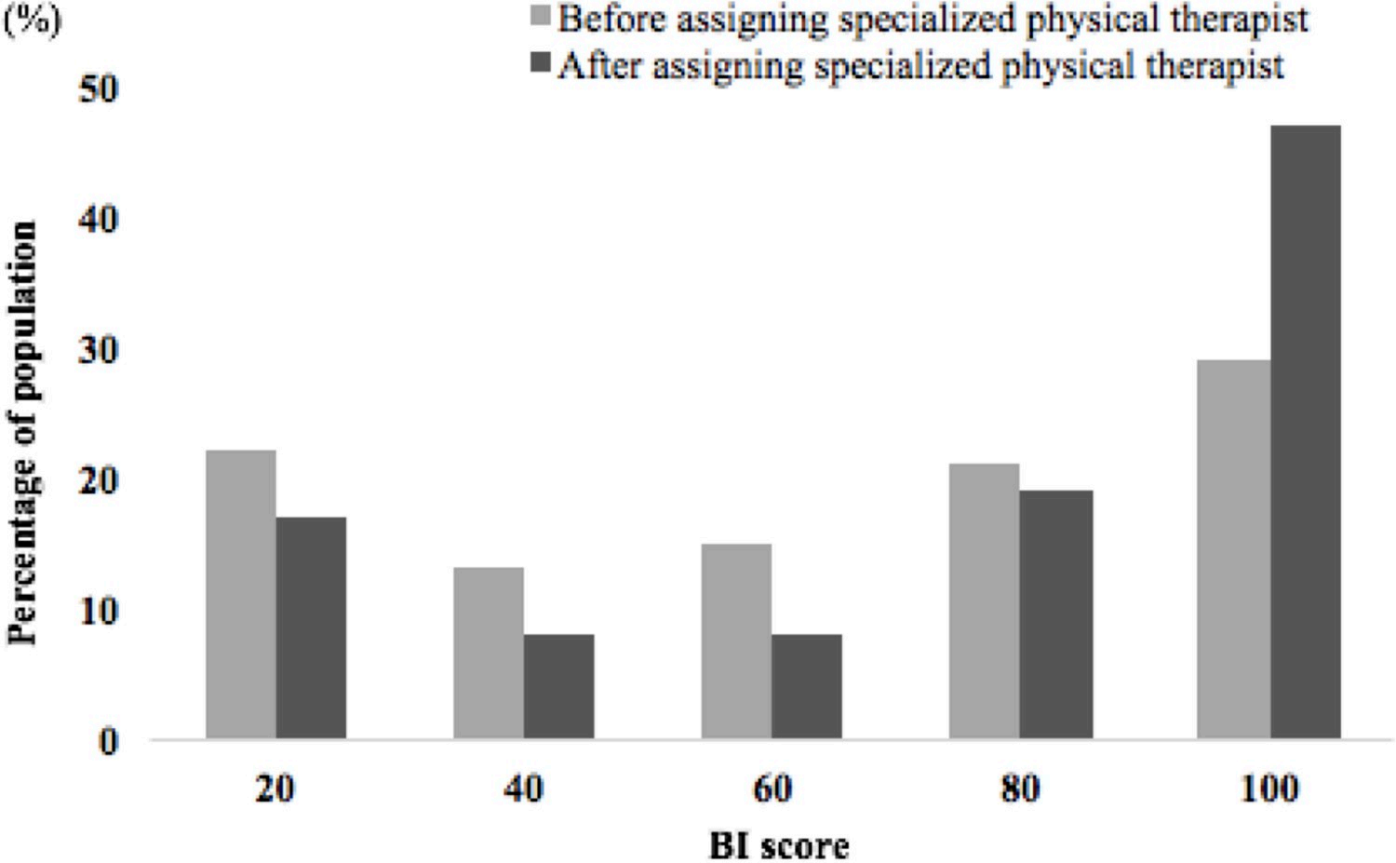
Distribution of patients with sepsis by BI score at hospital discharge.

The patients who underwent treatment with the specialized physical therapist had a significantly shortened period of days to begin the rehabilitation regimen compared to the control group (2.7 ± 1.6 days vs. 6.1 ± 5.8 days respectively, P<0.001, Fig 2). Table 1 compares the demographics of the treatment and control groups and shows significant differences in the DIC scores, medication use, BI at discharge, independent ADL rates, and length of hospital stay. Seventy-eight patients in the study returned to independent ADL at hospital discharge (independent ADL rate = 53.8%). Fig 3 shows the BI score distribution among the study patients at the time of hospital discharge.

**Table 1:** Demographic clinical characteristics in AECCC and hospital measures.

Variable	Before assigning specialized physical therapist (n = 86)	After assigning specialized physical therapist (n = 59)	*P-value*
The number of days until rehabilitation (days)	6.1 ± 5.8	2.7 ± 1.6	*P* < 0.001
Age (yr)	71.5 ± 13.8	68.1 ± 14.3	0.148
Men / women, n (%)	50 (58) / 36 (42)	35 (59) / 24 (41)	1.000
BMI (kg/m^2^)	21.2 ± 4.5	21.9 ± 10.9	0.540
GNRI score	81.3 (74.3, 86.2)	79.8 (71.5, 85.2)	0.178
Severity score			
SOFA score	5.6 ± 3.6	6.6 ± 3.3	0.051
SIRS score	2.6 ± 1.0	2.7 ± 0.9	0.396
DIC score	0 (0, 0)	1 (0, 4)	*P* < 0.001
PCT (ng/mL)	8.1 (0.9, 32.4)	16.7 (3.5, 49.7)	0.057
Primary source of infection			
Pneumonia, n (%)	8 (10)	10 (17)	0.265
Other plumonary disease, n (%)	6 (7)	1 (2)	0.288
Urinary tract infection, n (%)	19 (22)	15 (25)	0.791
Intra-abdominal infection, n (%)	8 (9)	9 (15)	0.406
Gastroenteritis, n (%)	18 (21)	7 (13)	0.232
Others, n (%)	27 (31)	16 (28)	0.712
Therapeutic medication			
Vasopressor, n (%)	35 (41)	47 (80)	*P* < 0.001
Steroid, n (%)	10 (12)	22 (37)	*P* < 0.001
Recomodulin, n (%)	11 (13)	18 (31)	0.016
Use MV, n (%)	30 (35)	20 (34)	1.000
Barthel index baseline	10 (0, 48)	5 (0, 33)	0.136
Outcome			
Barthel index discharge	63 (25, 85)	80 (40, 95)	0.018
Independent ADL, n (%)	39 (45)	39 (66)	0.022
Length of hospital stay (days)	28 (16, 46)	18 (10, 39)	0.016
Discharge to home, n (%)	41 (48)	32 (54)	0.44

Data are counts (percentages), mean ± SD or median (25%, 75%). Definition of abbreviations: AECCC indicates advanced emergency critical care center; BMI, body mass index; GNRI, Geriatric Nutritional Risk Index; SOFA, sequential organ failure assessment; SIRS, systemic inflammatory response syndrome; DIC, disseminated intravascular coagulation; PCT, procalcitonin; MV, mechanical ventilation; ADL, activities of daily living.

### Multivariate Logistic regression analysis

Table 2 shows the logistic regression analysis. According to the multivariable analyses, (1) assigning a specialized physical therapist (odds ratio = 2.40; 95% confidence interval = 1.09–5.79; P = 0.050) and (2) the number of days until rehabilitation (odds ratio = 0.24; 95% confidence interval = 0.08–0.76; P = 0.014) were significantly associated with the prevalence of return to independent ADL at hospital discharge.

**Table 2:** Logistic regression analysis of return to independent ADL at hospital discharge.

Variable	Univariable analysis		Multivariate analysis			
			Model 1		Model 2	
	OR (95% CI)	*P-value*	OR (95% CI)	*P-value*	OR (95% CI)	*P-value*
Assigning specialized physical therapist (Before vs After)	2.35 (1.18–4.67)	0.015	2.42 (1.11–5.78)	0.049	−	−
The number of days until rehabilitation (≤ 5 days vs ≥ 6 days)	0.35 (0.15–0.81)	0.014	−	−	0.25 (0.07–0.78)	0.016
Age (yr)	0.96 (0.93–0.98)	0.001	ND	ND	ND	ND
Men / women, n (%)	1.37 (0.70–2.66)	0.357	ND	ND	ND	ND
BMI (kg/m^2^)	0.99 (0.95–1.03)	0.585	ND	ND	ND	ND
GNRI score	1.11 (0.94–1.23)	0.252	ND	ND	ND	ND
Severity score						
SOFA score	0.87 (0.78–1.96)	0.006	ND	ND	ND	ND
SIRS score	0.92 (0.66–1.28)	0.617	ND	ND	ND	ND
DIC score	1.05 (0.92–1.20)	0.473	ND	ND	ND	ND
PCT (ng/mL)	1.00 (0.99–1.00)	0.487	ND	ND	ND	ND
Primary source of infection						
Pneumonia, n (%)	0.84 (0.31–2.26)	0.730	ND	ND	ND	ND
Other plumonary disease, n (%)	0.33 (0.06–1.74)	0.190	ND	ND	ND	ND
Urinary tract infection, n (%)	0.60 (0.28–1.30)	0.198	ND	ND	ND	ND
Intra-abdominal infection, n (%)	1.26 (0.45–3.52)	0.658	ND	ND	ND	ND
Gastroenteritis, n (%)	1.36 (0.57–3.26)	0.495	ND	ND	ND	ND
Others, n (%)	1.69 (0.81–3.50)	0.16	ND	ND	ND	ND
Therapeutic medication						
Vasopressor, n (%)	0.70 (0.36–1.36)	0.300	ND	ND	ND	ND
Steroid, n (%)	0.91 (0.84–0.99)	0.029	ND	ND	ND	ND
Recomodulin, n (%)	0.82 (0.38–1.80)	0.626	ND	ND	ND	ND
Use MV, n (%)	0.43 (0.21–0.86)	0.016	ND	ND	ND	ND
Barthel index baseline	1.00 (1.02–1.05)	*P* < 0.001	ND	ND	ND	ND
Length of hospital stay (days)	0.99 (0.97–0.99)	0.031	ND	ND	ND	ND
Propensity score	ND	ND	212 (41–1100)	*P* < 0.001	238 (45–1270)	*P* < 0.001

Multivariable analysis indicates the adjusted effect by applying propensity score which is a conditional probability given by other clinicopathologic factors including age, sex, BMI, GNRI score, SOFA score, SIRS score, DIC score, PCL, primary source of infection, vasopressor, steroid, recomodulin, use MV, barthel index baseline, length of hospital stay. Definition of abbreviations: OR, odds ratio; CI, indicates confidence interval; ND, not done; BMI, body mass index; GNRI, Geriatric Nutritional Risk Index; SOFA, sequential organ failure assessment; SIRS, systemic inflammatory response syndrome; DIC, disseminated intravascular coagulation; PCT, procalcitonin; MV, mechanical ventilation.

## Discussion

This study found that sepsis patients who received early rehabilitation with a specialized physical therapist had a significantly better return to independent ADL, BI at discharge, and shortened length of hospital stay. This suggests that treating sepsis patients by a specialized physical therapist improved rehabilitation awareness in the AECCC, increased rehabilitation requests, and enrichment team medical care. Logistic regression analysis showed that treatment by the specialized physical therapist and the number of days until rehabilitation after hospital admittance also predicted the return to independent ADL in sepsis patients. To our knowledge, this is the first study to examine sepsis patients and discuss the relationship between early rehabilitation by a specialized physical therapist and the return to independent ADL. As opposed to previous studies that examined the relationship between long-term rehabilitation and ADL recovery for septic patients [15], this study looked at short-term rehabilitation and ADL recovery. The physical therapy treatment at our center focused primarily on physical rehabilitation, which is the function measured by the BI.

In Europe and the United States, early mobilization is defined as physical activity that occurs within two to five days after admittance to a hospital in Europe and America [23, 24]; this is the period in which muscle mass degenerates due to immobility onset of disease, surgery, or acute exacerbation [26]. In this study, the duration time between admission and rehabilitation intervention by the specialized physical therapist was 2.7 ± 1.6 days, which can be considered to indicate that early rehabilitation was successful. In scientific studies, physical exercise for patients with sepsis has been found to improve physical function. In animal models of sepsis, physical exercise increased bacterial clearance from blood and organs, decreased the release of pro- and anti-inflammatory cytokines, and improved survival [27].

Assigning a specialized physical therapist is one method for reducing the number of days until rehabilitation begins, and it is easy to adopt in many hospitals. Furthermore, changes in vital signs during the rehabilitation therapy and laboratory data suggest that early rehabilitation therapy could be safely performed in sepsis patients who required intensive care or had worsening complications. However, in AECCC facilities without adequate numbers of well-trained staff, this level of rehabilitative support might be difficult.

This study’s results show that introducing early rehabilitation provided by a specialized physical therapist not only improved the BI score at discharge but also significantly increased return to independent ADL and shortened the length of hospital stay. In previous studies, one third of patients with sepsis had not returned to independent living after six months [28]. While the number of critically ill patients who survive after admittance to AECCC and ICU has increased, the number of patients who do not regain ADL at the time of discharge has also increased. If these patients need to go to a long-term rehabilitation center after hospital discharge, they face increased medical expenses and a strain on medical resources. They can also experience physical deterioration, which may lead to further complications and the necessity for hospital readmission [8–10, 28, 29]. Therefore, this study suggests that overall medical costs can be reduced by increasing the speed of return to ADL and shortening the length of hospital stay. However, there was no significant difference in the discharge to home. Most likely, this is because the family environment and social background of the patients influenced their discharge decisions.

Rehabilitation using EMS was performed for patients with difficulty in sitting up in bed and transfer before early rehabilitation was provided by a specialized physical therapist. The physiological effect of EMS on ICU patients, including those with sepsis, has been shown in previous studies; concentrated EMS in the ICU also induced systemic benefits on the microcirculation, which is closely related to endothelial function [30]. In our study, patients in both groups received EMS, so its individual effect is unknown, especially for subjects with difficulty leaving their beds. Introducing EMS in early rehabilitation can minimize ICU-AW.

The demand for early and intensive rehabilitation is increasing, especially in the fields of emergency and intensive care medicine. For effective and safe rehabilitation in the emergency room or ICU, there must be information sharing and discussion among the hospital’s medical staff members. Hospitals must commit to adding a specialized physical therapist for their critically ill patients in the emergency units and ICUs. Further research can demonstrate the importance of having this specialized therapy available to allow sepsis patients to return to independent ADL.

This study had limitations. First, we only included patients from a single center, which limits the validity of the study. Second, we did not assess cognitive or emotional status to evaluate the effects of this specialized physical therapy and the importance of returning to independent ADL after sepsis. Finally, details about the rehabilitation program and the effect of the amount of time spent with each patient are not discussed. Future clinical studies should include patients from multiple centers, and more details about the patient’s pre-hospitalization.

## Conclusion

Assigning specialized physical therapists for sepsis patients at our AECCC significantly shortened the number of days until rehabilitation began after the patient was admitted to the hospital and improved the ADL at hospital discharge. Multivariate logistic regression analysis showed that the two factors predicting an independent ADL in sepsis patients were (1) assigning a specialized physical therapist and (2) the number of days between hospitalization and the beginning of rehabilitation. We that recommend this method will be easy to adapt for other centers and will be effective for their patients with sepsis. This study revealed that assigning a physical therapist as part of early rehabilitation may be more effective in achieving ADL independence in sepsis patients.

## Supporting information

S1 File (XLSX)
